# Longitudinal study of Good Pharmacy Practice roles covered at the annual world pharmacy congresses 2003–2019

**DOI:** 10.1186/s40545-022-00482-4

**Published:** 2022-11-28

**Authors:** Zuzana Kusynová, Hendrika A. van den Ham, Hubert G. M. Leufkens, Aukje K. Mantel-Teeuwisse

**Affiliations:** 1 International Pharmaceutical Federation (FIP), The Hague, The Netherlands; 2 Utrecht WHO Collaborating Centre for Pharmaceutical Policy and Regulation, Division of Pharmacoepidemiology and Clinical Pharmacology, Utrecht Institute for Pharmaceutical Sciences (UIPS), Utrecht University, PO Box 80082, 3508 TB Utrecht, The Netherlands

**Keywords:** Pharmacy, Good Pharmacy Practice (GPP), Congress programmes, Pharmacy profession, Evolution

## Abstract

**Background:**

Globally accepted roles of pharmacists are described in the Good Pharmacy Practice (GPP) standards, published by the World Health Organization (WHO) and the International Pharmaceutical Federation (FIP) in 2011. These standards provide a wide-ranging description of four main roles pharmacists fulfil. The global platform, where pertinent discussions around excellence and innovation in various pharmacy roles take place, is the annual congress of the pharmacy organisation representing the profession globally, FIP.

**Objectives:**

Given the world pharmacy congresses present and reflect on the most topical and contemporary matters, this longitudinal study aimed at creating a historical overview of the frequency of appearance of the different GPP roles in the programmes of the past 17 congresses (2003–2019). This is to distinguish the dominance of different roles over time and thus their relevance for the profession.

**Methods:**

The GPP standards served as a framework to create a set of keywords that were analysed for their frequencies of appearance in the programmes through text analysis. Trends in the four overarching GPP roles and at individual keyword level were analysed descriptively over time.

**Results:**

The study found that all four GPP roles appeared in the programme each year and none of them was significantly missing, neither in the decade preceding the publication of the GPP standards nor in the decade thereafter. Role 3 “Maintain and improve professional performance” was most frequently represented, also demonstrating an upward trend in appearance, together with Role 4: “Contribute to improve effectiveness of the health-care system and public health”. Trends emerged towards patient-centred clinical focus and positioning pharmacy as an important player in the health-care system—observed also at individual keywords level in areas such as health promotion—away from the more traditional product-centred practice roles such as compounding.

**Conclusions:**

GPP roles have been already covered by the FIP annual congresses (long) before 2011, when the GPP roles were formally adopted, and they stayed relevant in the decade after. The more pronounced dominance toward the roles related to improving professional performance and positioning pharmacy are in line with the trend that the rather technical topics in pharmacy are increasingly covered by specialised meetings and that the FIP annual congresses have moved toward more general, scholarly platforms for dialogue and conversation.

## Background

The World Health Organization (WHO) and the International Pharmaceutical Federation (FIP) have clearly described the significant role of pharmacists in the increasing health demands [1]. In the past 20 years, the society’s expectations towards health care have changed. The changes in regulation of medicines led to greater accessibility to quality assured medicines. Supplying medicines alone, however, is not sufficient to achieve the desired treatment outcomes. Promoting medication adherence and minimising potential adverse effects have become an integral part of good dispensing practices [2]. The ever-growing and complicated variety of medications and non-adherence to prescribed medications have compelled the pharmacist’s position to evolve into a more patient-centred health-care professional (e.g. pharmaceutical care giver) [3]. Pharmacists nowadays have a greater responsibility to handle all the medication-related needs and to help patients to make the best use of the medicine while at the same time treatments have become complex (e.g. a shift from small molecules to biologicals or individualised treatment). They are not only expected to address acute health needs, but also to address the burden of chronic diseases and promote wellness [3, 4]. Finally, as an integral health-care team member, pharmacists collaborate with other members in the health system and contribute to its efficiency. Together with the prescriber, they are responsible for the health outcomes of the patient [3, 5].

Pharmacists are described as sensitive to the needs of the society and the profession has a potential to evolve its roles accordingly [6]. The globally accepted roles of pharmacists are described in the Good Pharmacy Practice (GPP) standards, published in 2011 jointly by the WHO and FIP. The GPP standards are intended to encourage national pharmaceutical organisations around the globe to focus the attention of community and hospital pharmacists on translating the roles described into the practice they provide [1]. These standards provide a wide-ranging description of four main roles pharmacists fulfil, in all settings, but especially community and hospital pharmacy settings. Each of the four roles is supported by respective functions that pharmacists fulfil, to respond to the health needs of the people through optimal, evidence-based care [1].

The GPP standards are still considered valid standards of today. However, over the past two decades, a number of factors have directly or indirectly contributed to evolution of pharmacy practice roles [6]. Therefore, questions arise whether the GPP roles described are still relevant, if they have been equally covered in past decade(s), and whether any of the roles became more prominent than others, or whether any roles diminished. The global platform, where pertinent discussions around excellence and innovation in various pharmacy roles in all settings takes place, is the annual congress of the pharmacy organisation representing the profession globally, FIP [7]. Here, pharmacists and pharmaceutical scientists present and reflect on the most topical professional and scientific activities at that time. As such these congresses act as a ‘mirror’ of what’s going on, of what’s hot, of what should be addressed and discussed, keeping in mind all the regional and national variation in priorities and cultural flavours.

In order to observe the evolution of different pharmacy roles, this study aimed at creating a historical overview of the frequency of appearance of the different roles of the pharmacists in the programme of the past nearly two decades. The study examined whether GPP roles described were already being reflected in the programme in the decade preceding their publication and also whether any of the roles became more prominent than others, or whether any roles diminished in the decade after their publication, indicating a gap. This is to distinguish the dominance of different roles over time and thus their relevance for the profession.

## Methods

### Pharmacy practice roles’ framework selection

The Good Pharmacy Practice (GPP) standards [1], which contain a comprehensive, internationally recognised framework of pharmacy practice roles as standards for quality of pharmacy services, served as a framework for the analysis. The GPP standards outline four key roles for pharmacists. Role 1: Prepare, obtain, store, secure, distribute, administer, dispense and dispose of medical products; Role 2: Provide effective medication therapy management; Role 3: Maintain and improve professional performance; and, Role 4: Contribute to improve effectiveness of the health-care system and public health. Each Role is supported by respective Functions that pharmacists fulfil in their practice. These are described in Table 1.

**Table 1:** Keywords framework based on Good Pharmacy Practice standards

Keywords framework based on GPP			Average number of appearances per year
Role 1. Prepare, obtain, store, secure, distribute, administer, dispense and dispose of medical products			
Function 1A: Prepare extemporaneous medicine preparations and medical products			8
Role characterised by keyword	Keywords including synonyms and related words	Search terms used	
Compound	Compounding	compound*; extemporaneous	4
(Ensure) quality of medicines^†^	Quality of medicines	quality*	16
Prepare (drug) formulation^†^	(Drug) Formulation	formulat* (ion)	3

Function 1B: Obtain, store and secure medicine preparations and medical products			7
Role characterised by keyword	Keywords including synonyms and related words	Search terms used	
Ensure access (to medicines)	Access (to medicines)	access*	9
Store medicines properly / ensure proper storage (conditions)	Storage (conditions); stability (of medicines)	stor*; stabili*	1
Procure (medicines)	Procurement (of medicines)	procure*	1
Select (medicines) ^†^	Selection (of medicines)	select*	1
Manage shortage (of medicines)	Shortage; stock-out	shortage; stock*	4
Manage controlled substances	Controlled substances; opioid (medicines)	control*; opioid*	1
Prevent substandard and falsified medicines	Substandard and falsified (medicines); substandard (medicines); substandard/spurious/falsely labelled/falsified/counterfeit (medicines); SF (medicines); SSFFC (medicines) Fake (medicines); adulterated (medicines) Unlicensed (medicines) Counterfeit	substandard and falsified; substandard; falsif*; spurious; SF; SSFFC; fake; adulterate*; unlicensed; counterfeit*	10
Ensure medication / patient safety/safety of medicines^†^	(Safeguarding) safety (of medicines); patient safety; reporting (errors)	safe*; safety*; patient safety; report*	29

Function 1C: Distribute medicine preparations and medical products			8
Role characterised by keyword	Keywords including synonyms and related words	Search terms used	
Supply (medicines)	Supply (of medicines); distribution (of medicines)	supply*; distrib*	12
Supply essential medicines	Essential medicines	essential*	2
Ensure disaster or pandemic (preparedness)	Disaster (response); emergency (response/ preparedness); humanitarian (environment)	disast*; emergency; humanit*	14
Supply new medicines	New medicines (& drugs & therapies)	new*	4
Monitor adverse events and safety issues	Reporting adverse events; pharmacovigilance	report*; vigilan*	8

Function 1D: Administration of medicines, vaccines and other injectable medications			2
Role characterised by keyword	Keywords including synonyms and related words	Search terms used	
Administer (medicines)	Administration (of medicines)	admin*	2
Immunise / Vaccinate	Immunisation; Vaccination	vaccinat*; immuni*	5
Participate in directly observed therapy (DOT) programmes	Directly observed therapy (DOT) programmes	directly observed therapy (DOT)	0
Manage infectious diseases^†^	Infectious / communicable diseases (management)	infectio*; communicable	3

Function 1E: Dispensing of medical products			2
Role characterised by keyword	Keywords including synonyms and related words	Search terms used	
Dispense (medical products)	Dispensing (practices)	dispens*	5
Counsel^†^	Counselling	counsel*	3
Document / assure documentation	Documentation (practices)	documentation	1
Evaluate prescription (of medicines)	Prescription (of medicines)	prescription	1
Evaluate electronic prescription (of medicines) ^†^	Electronic prescription (of medicines); e-Health	e* -prescription; eHealth; e-Health	1
Perform generic substitution	Generic substitution	generic*	0
Ensure (patient / consumer) confidentiality	Patient data; (patient or consumer) confidentiality	confidential*; data*	4

Function 1F: Dispose of medicine preparations and medical products			3
Role characterised by keyword	Keywords including synonyms and related words	Search terms used	
Dispose (medicines)	(Medicines) disposal; (medicines) waste (disposal); environmental impact	dispos*; wast*; environment*	6
Monitor inventory	Inventory	inventory	0

Role 2. Provide effective medication therapy management			
Function 2A: Assess patient health status and needs			2
Role characterised by keyword	Keywords including synonyms and related words	Search terms used	
Assess health and needs (of patient or consumer)	(Individual) (patient or consumer) assessment (of health and needs)	assess*	1
Use Electronic Patient Health Record (for health assessment) ^†^	Electronic Patient Health Record	Electronic Patient Health Record	1
Assess health literacy	(Health) literacy (assessment)	litera*	4

Function 2B: Manage patient medication therapy			8
Role characterised by keyword	Keywords including synonyms and related words	Search terms used	
Assure rational use (of medicines)	Responsible use of medicines; rational use of medicines; appropriate use of medicines; prudent use of medicines; quality use of medicines; cost-effective use of medicines	responsible use; rational use; appropriate use; prudent use; quality use; cost-effective use*	4
Assure cost-effectiveness (of medicines)	Pharmacoeconomics; pricing (of medicines); cost of medicines; cost of care	economic*; pric*; cost*; cost*	14
Observe treatment guidelines	Treatment guidelines; therapeutic guidelines	treatment guid*; therapeutic guid*	1
Select appropriate medication dosage	(Medicine) dosage; individualised therapy; personalised therapy; drug delivery systems; drug absorption; bioavailability/bioequivalence; pharmacokinetics and pharmacodynamics (PK/PD)	dosage*; personalised therapy; individualised therapy; (drug) delivery; (drug) absor*; bioavailab*; *kinetic*	21
Coordinate medication therapy management	Medication/Drug Therapy Management; MTM	* therapy management; MTM	1
Monitor (therapy)	(Therapy) monitoring	monitor* (therapy)	1
Improve adherence^†^	Adherence; compliance	adher*; complian*	17
Ensure continuity of care	Continuity of care; Transition of care across health-care system; Transfer (of care); Transfer (of information); (Patient) referral (to other health-care professional);	continuity (of care) transit* (of care) transfer* (of care) transfer* (of information) referral + refer* (context of referring patients)	1
Manage noncommunicable diseases^†^	Noncommunicable diseases (management); NCDs; chronic (diseases management)	non-communicable*; non communicable*; noncommunicable*; NCD*; chronic*	4

Function 2C: Monitor patient progress and outcomes			14
Role characterised by keyword	Keywords including synonyms and related words	Search terms used	
Monitor outcomes	Outcomes monitoring	outcome	22
Assess drug utilisation^†^	Drug utilisation	utili*	1
Consider patient diagnosis^†^	Patient diagnosis	diagnos*	5

Function 2D: Provide information about medicines and health-related issues			13
Role characterised by keyword	Keywords including synonyms and related words	Search terms used	
Provide information (medicine / health related)	(Medicine / health) information	info* (medicine / health)	30
Empower (patient or consumer)	(Patient or consumer) empowerment; Consultation (with patient or consumer)	empower*; consult*	3
Reduce antimicrobial resistance	Antimicrobial (or antibiotic) resistance	resistan*	6

Role 3: Maintain and improve professional performance			
Function 3A: Plan and implement continuing professional development strategies to improve current and future performance			15
Role characterised by keyword	Keywords including synonyms and related words	Search terms used	
Adopt continuing professional development strategies	Continuing education; continuing professional development	educat*; Continuing Professional Development	10
Implement workforce development strategies^†^	Workforce (development) Human resources (development)	workforce human resource*	13
Update knowledge and skills about complementary therapies	Complementary therapies; alternative therapies; herbal therapies; traditional Chinese therapies; Supplements; homeopathy	complementary; alternative*; herbal*; Chinese* (therapies); supplement*; homeopath*	10
Update knowledge on new technologies	New technologies; biotechnology; digital technology	technolog*; biotech*; digital*	25

Role 4: Contribute to improve effectiveness of the health-care system and public health			
Function 4A: Disseminate evaluated information about medicines and various aspects of self-care			5
Role characterised by keyword	Keywords including synonyms and related words	Search terms used	
Contribute to improved effectiveness of the health system	Health system (strengthening)	health system*	3
Contribute to improved public health	Public health	public health*	10
Improve self-care	Self-care; self care; selfcare	self-care; self care; selfcare	1
Assure evidence-based care	Evidence-based (care)	evidence-based	4
Provide care to patient populations with wide range of age groups	Age population; children; paediatric; elderly (therapy)	age; child*; paediatr; * elderly	9
Provide care to patient populations with wide range of health literacy levels	Health literacy (improve)	litera*	4
Advise on Internet-obtained information	Internet; social media	Internet; social media	4

Function 4B: Engage in preventive care activities and services			2
Role characterised by keyword	Keywords including synonyms and related words	Search terms used	
Engage in health promotion	Health promotion; (disease) prevention; preventive care	health promot*; preventi*; preventive care	5
Engage in harm reduction^†^	Harm reduction; drug abuse	harm*; abus*	0
Provide smoking cessation	Smoking cessation	smok*	1
Provide point-of-care testing	Point-of-care testing	point-of-care*	1
Provide screening	Screening	screen*	2

Function 4C: Comply with national professional obligations, guidelines and legislations			9
Role characterised by keyword	Keywords including synonyms and related words	Search terms used	
Comply with the provisions of a national standards of ethics	Code of ethics; ethics; oath	ethic*; ethic*; oath	8
Comply with accreditation standards^†^	Accreditation standards	accredit*	1
Comply with regulation / standards^†^	Regulation; harmonisation; Pharmacopeia	regulat*; harmoni*; harmoni*	19

Function 4D: Advocate and support national policies that promote improved health outcomes			8
Role characterised by keyword	Keywords including synonyms and related words	Search terms used	
Advocate (for improved health outcomes)	Advocacy	advoca*	4
Support health-care policies	Health-care policies	polic*; policy-makers + policy makers	14
Collaborate with other health-care professionals	Interprofessional collaboration	collaborat*	13
Ensure integrated care^†^	Integrated care	integrated care	1

^†^The explicit wording under respective GPP role was adjusted

### Keywords identification and categorisation

The GPP standards were scrutinised with the objective to identify the keywords representing each of the GPP Roles and respective Functions. For the keywords either the exact wording in the description of the role was used, or if not deemed suitable the wording was adjusted by the researchers. The keywords were categorised under each Role and respective Function based on the judgement of the researcher (ZK) and independently validated by a second researcher (HAvdH). In case of doubt, the final choices were selected based on a discussion between the researchers.

The final framework (see Table 1) contained only the keywords that were comprehensive, clearly characterising the pharmacy practice Role and respective Function. Seventy keywords were selected for the text analysis. The keywords were complemented with synonyms and/or equivalent words under each keyword. For example, counterfeit medicines was the preferred term used mostly until 2011, replaced by the official term SSFFC (substandard, spurious, falsely labelled, falsified, and counterfeit) in 2012–2017 and substandard and falsified (SF) medicines used after 2017 [8]. Adding synonyms or equivalent and related words from the same area led to a total of 142 keywords.

### Congresses programmes selection

The appearance of GPP roles in the annual congress programmes of FIP were analysed for the period of the past 17 years (2003–2019), as these programmes are available in an electronic format and thus suitable for analysis. Moreover, this timespan covers 8 years before the formal introduction of the GPP roles and 8 years thereafter, covering nearly two decades altogether. The congresses of 2020 and 2021 were cancelled due to COVID-19 pandemic, therefore excluded from the analysis.

These annual pharmaceutical congresses organised by FIP, called the World Congress of Pharmacy and Pharmaceutical Sciences, were chosen because they are the biggest global annual congress for pharmacists. The programme is comprehensive and touches on a wide variety of areas of pharmaceutical practice and importantly, is aligned with the input and priority topics of the largest global network of national pharmacy organisations. The congresses attract a rather stable audience mainly through the international membership. They are managed by the longstanding staff under long-term quality standards, in a uniform venue, format and timing/term. Other conferences or events organised by FIP were excluded from the analysis.

### Data analysis

The keywords were used for text analysis of the congress programmes, and the frequency of appearance of keywords was counted under each Role and Function. Both identification of keywords in the GPP document and text analysis of the congress programmes were performed using MS Excel and Adobe Reader. The number of keyword synonyms and/or equivalent words were manually counted under each Role and respective Function as they appeared. Another researcher (HAvdH) validated this step by counting the frequencies of keywords from a random sample (*n* = 10%) of programmes independently. No inconsistencies were found.

Keyword frequencies were summed up and the averages were calculated for comparison under each Function. Average of the Functions’ frequencies was calculated for each Role. Roles were compared in a graphical overview with trendlines displayed (linear regression R^2^ value was calculated to assess variability in the trend) and average annual growth change was calculated for comparison for each Role. To account for the difference in total number of keywords per year, the relative distributions of the four roles over the years were displayed in a stacked bar chart.

In addition, an average of keyword appearance over the years was calculated to find the most frequent keywords. The standard deviation was calculated to reflect the width of data distribution, for illustration. Lastly, the frequencies were colour coded based on a threshold (top 10%) to highlight peaks of appearance and facilitate trend identification. For these keywords where trends were concluded, linear regression R^2^ value was calculated to assess variability in the trend.

## Results

**Fig. 1:**
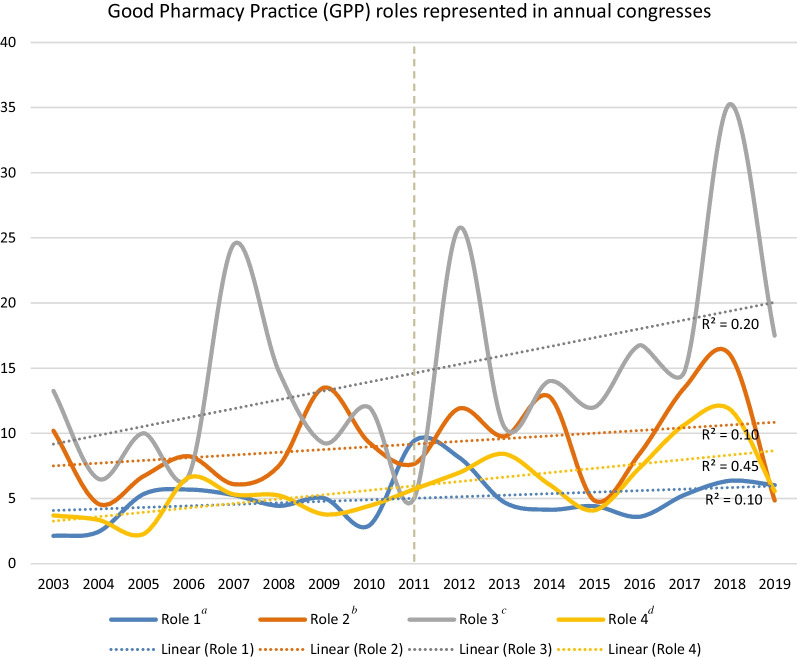
Good Pharmacy Practice (GPP) roles represented in annual congresses. ^a^Corresponding to *n* = 6 Functions; ^b^Corresponding to *n* = 4 Functions; ^c^Corresponding to *n* = 1 Functions; ^d^Corresponding to *n* = 4 Functions; Role 1: Prepare, obtain, store, secure, distribute, administer, dispense and dispose of medical products; Role 2: Provide effective medication therapy management; Role 3: Maintain and improve professional performance; and, Role 4: Contribute to improve effectiveness of the health-care system and public health

All GPP roles were reflected in each of the programmes at least once, already in the decade preceding their publication, and also in the decade after their publication. Table 1 lists the average appearances per year and Fig. 1 outlines the annual frequencies of the Roles’ appearance corrected for the number of functions, and their respective trends.

Role 3: Maintain and improve professional performance was represented most frequently in all years except three, with the highest peak in 2018. Role 2: Provide effective medication therapy management was the second most represented except for 2019, when it was the least represented Role, and in 2009, where it was the most represented Role. Roles 4: Contribute to improve effectiveness of the health-care system and public health and Role 1: Prepare, obtain, store, secure, distribute, administer, dispense and dispose of medical products were represented steadily but with lower frequencies. Looking at the trends, all Roles demonstrated an upward trend. Role 4: Contribute to improve effectiveness of the health-care system and public health demonstrated the highest average annual change (6.3%), followed by Role 3 (5.0%). For Role 1 and Role 2 the annual increase was 2.4% and 2.3%, respectively.

**Fig. 2:**
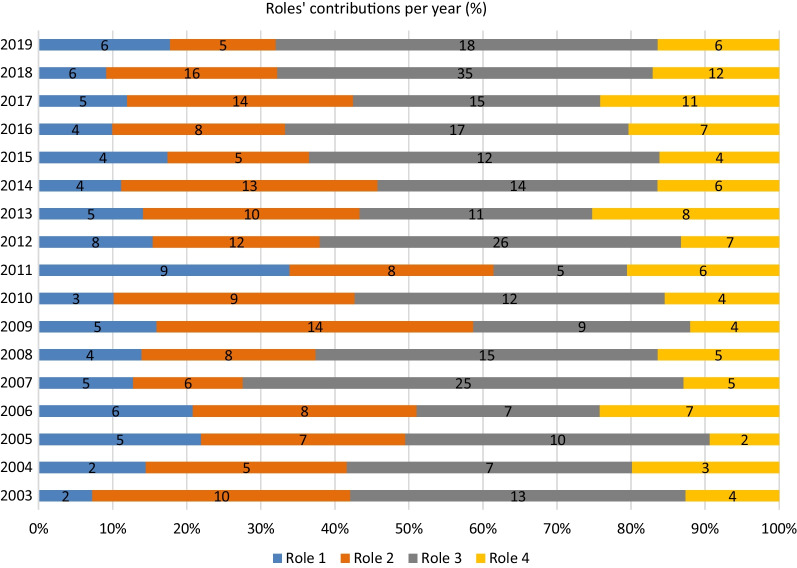
Roles’ relative contribution (%) to the congress programme

Figure 2 depicts the relative distributions of the four roles over the years. The percentage contribution of each of the roles shows a rather homogeneous distribution over the years, with dominance of Role 3 in all year except 1 year 2009.

**Fig. 3:**
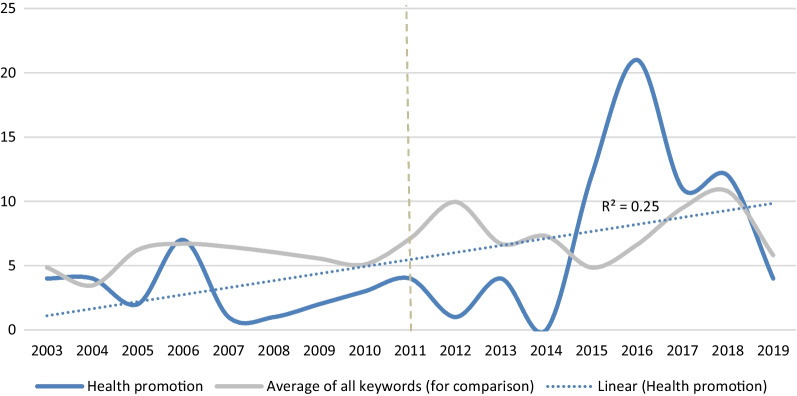
Appearance of keywords related to health promotion by pharmacist

Looking at individual keywords, all the keywords (*n* = 70, 100%) appeared in at least one congress, while most of the keywords (*n* = 61, 87%) appeared in each of the congresses. When looking for upward trends, a theme that clearly emerged is responsibilities related to health promotion and preventive activities and services, with a spike after 2015 (Role 4); see Fig. 3. Another example of a theme with upward trend is collaboration with other health-care professionals, a theme under Role 4 that clearly emerged after 2005, with major spikes in 2008, 2010 and 2013.

**Fig. 4:**
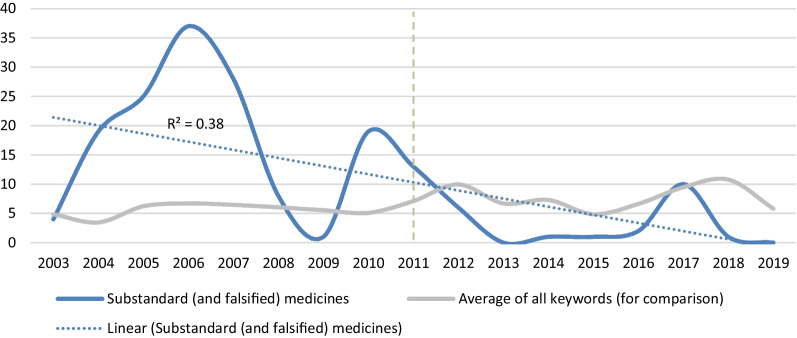
Appearance of keywords related to minimising substandard and falsified (SF) medicines

On the other hand, there were keywords that diminished in frequency. One downward trend identified was for keywords related to preventing substandard and falsified (SF) medicines (average = 10, SD = 11.1, highest peak *n* = 37 in 2006, Role 1); see Fig. 4. Downward trends were further identified in keywords related to reducing antimicrobial resistance (average = 6, SD = 6.0, highest peak *n* = 22 in 2010, Role 2) and activities improving self-care (average = 1, SD = 2.8, highest peak *n* = 12 in 2007, Role 4).

There were also keywords that were mentioned in certain periods more than in others with no conclusive upwards or downwards patterns. For example, compounding (Role 1) appeared on average 4 times per year (SD = 6.6) with peaks in 2009 (*n* = 21) and in 2013 (*n* = 20), with no clear time trend.

## Discussion

This longitudinal study aimed at creating a historical overview of trends in the appearance of the different pharmacy practice roles, as defined by the GPP standards, in the programme of the past 17 world pharmacy congresses (2003–2019). The study found that all the four roles were already being reflected in all programmes in the decade preceding their publication (2011), and also in the decade after their publication. This is pointing out that these Roles were relevant for the profession already before the GPP were published and stayed relevant even long after their publication. They were nonetheless not equally covered in the selected period, with Role 3 (Maintain and improve professional performance) being the most frequently represented. Under this Role, pharmacists plan and implement continuing professional development strategies to improve their current and future performance. As society demands better, innovative care, pharmacists need to keep themselves abreast on current events and attend formal lifelong learning systems (e.g. continuing education) to sustain their competences and ensure the provision of quality patient care [3]. The aim of the world congress is also to fulfil this role. Moreover, in 2016, education has been formalised as an important part of pharmacy, complementing practice and science [7, 9]. Many discussions have preceded this formalisation as well as the important role of education in facilitating the full implementation of clinical pharmacy. [10, 11]

Role 3 was also the role with a clear upward time trend, together with Role 4: Contribute to improve effectiveness of the health-care system and public health. For example, under this role pharmacists are involved in public health through delivery of health prevention programmes. Both roles 3 and 4 position pharmacy in a vital place in the health system, where practice is underpinned by the willingness to increasingly provide patient-centred services. Such positioning is accompanied by discussions focused on embracing innovative services by pharmacists—by education and through public health advocacy [12–14], corresponding back to Roles 3 and 4. Although high-income countries are debating futuristic approaches, clinical skills, and expanding pharmacy services for these Roles, a large majority of low-to-middle-income countries still lag behind in strengthening pharmacy practice [15, 16]. In comparison, product-focused Role 1 may not have sparked as many discussions, given it is considered business-as-usual in dispensing and administering of medicines. Indeed, the upward trend in Role 1 and 2 was much milder.

The official publication of GPP may be seen as an important milestone, when the profession agreed on the global standards both internally within pharmacy (from FIP’s side) and externally within health care (from WHO’s side) [1]. What we observed is that after the publication, in the immediate congress that followed in 2012, there was a sharp increase in the frequency of appearance of Role 3, and a mild increase in Role 2 and Role 4, while Role 1 decreased. This is similar to the observed overall long-term trends described earlier, and in line with the notion that patient-centred roles sparked more discussion than the product-focused role.

### Individual keywords

One of the strong examples of an increase of appearance of new services that bring pharmacists closer to the patient is health promotion (Role 4). These are activities by pharmacists related to disease prevention and preventive care. Health promotion itself as a concept was defined in 1998 [17], however only in 2016 it was linked to the UN Sustainable Development Goals [18]. Pharmacists were already advocating for recognition at global level ahead of this milestone [19–21] and the frequent appearance of this theme in 2015 was related to the key joint session with the World Health Organization (WHO) on health promotion activities by pharmacists. These were later highlighted by the strengthening of the position of pharmacists in the global primary health-care agenda [22–24] as well as in the congress programmes.

Another example is collaboration with other clinical care professionals (Role 4), a theme that clearly emerged after 2005, with major spikes in 2008, 2010 and 2013. The spike in year 2010 coincides with the WHO’s global action framework [25] in this area, further supported by the 2013 joint statement of the World Health Professions Alliance (WHPA) strengthening the collaboration of dentists, nurses, physical therapists, physicians and pharmacists [26]. Literature was long criticising the siloed approach, that is now shifting towards improved collaboration in the last decade. [26–28]

Examples above illustrate that pharmaceutical practice/care as a concept has moved the pharmacy profession from primarily focusing on the product (the medicine itself) to the clinical area of patient's therapy and how it should be optimised for the individual patient [9]. Pharmacists irrespective of their work setting interact and provide direct patient care as a clinical service, that has established its role within the society and the health-care system. [29] On the other hand, traditional roles centred around the product such as compounding were sporadically featured, as a rather closed topic on the international agenda. A further research could examine the reasons for dominance of each of the Roles, e.g. through qualitative studies linking the findings of this study with overall trends in pharmacy.

Some of the topics that were prominently featured in the past diminished in recent years. Examples include the minimising of substandard and falsified medicines, reducing antimicrobial resistance or promoting self-care. They did not appear at the congresses but are discussed more at strategic level—led by international organisations (e.g. in collaboration with global bodies, such as WHO). This may be caused by the fact that in order to progress on these different roles (e.g. in minimising AMR), more discussion is needed both within pharmacy but also with a broader set of players in the health-care system [30–32]. Some of these topics may then re-appear at the congress in the future or become institutionalised. This analysis may help the balancing of the agenda and programme planning and feed into strategic planning.

Our finding that all the four GPP roles were being well reflected in the programmes of the FIP annual congresses should not be taken as a surprise. The GPP roles are the heart of the Federation, as the annual congresses are. Both can be seen as two sides of the same coin. What this study adds and makes interesting is twofold. First, the GGP roles, both literary and in terms of context have been already covered by the FIP annual congresses (long) before 2011, when the GGP roles were formally adopted. So FIP annual congress may have acted as nurseries, as sandboxes so to say, for the ideas, directions, and philosophy for the GPPs under development. Second, the prominent appearance of the roles related to improving professional performance and positioning pharmacy, i.e. most non-technical roles, are in harmony with the trend that the FIP annual congresses have moved toward educational platforms for dialogue and conversation, while the technical topics in pharmacy are increasingly covered by expert meetings.

### Limitations

There are several limitations to note in this study. Firstly, the final keywords selection and selection of synonyms and equivalent and/or related words representing GPP framework was based on authors’ judgement, although validated by teamwork of two researchers. In addition, the study did not intend to find keywords covered in the programmes that fell outside of the GPP standards. Secondly, the FIP congresses programmes, while taking into consideration the input from its global membership and being selected by an independent programme committee, may not thoroughly cover all topics pertinent for pharmacy practice. While the main congress audience is pharmacy practitioners, smaller but considerable part of the audience is composed of pharmacists working in pharmaceutical science, academia, or industry. Therefore, choosing what will feature on the agenda may lead to exclusion of certain topics. In addition, the study focused solely on pharmacy practice related topics and excluded pharmaceutical sciences topics while these could also have been discussed at the congress, however outside of the scope of this study. Similarly, some activities may not yet be described by the GPP and therefore were potentially not captured by this study, given that pharmacy practice continues expanding in different countries or jurisdictions all over the world [2, 6, 29, 33]. For example, in some jurisdictions pharmacists assume the authority to prescribe medications for minor ailments in defined situations [34] or they perform de-prescribing (e.g. in elderly patients with multiple treatments) [35]. While examples above do not seem to be robust enough to form another Role as such, they could be included under some of the existing ones. Lastly, while we observed some changes in the frequency of appearance of the different GPP roles before and after their official publication in 2011, a more detailed quantitative analysis is not possible given uncertainty around the real length of the consultation process. Despite these limitations, the methods provided a rich depth of information and promoted trustworthiness of findings and clear and consistent themes emerged.

## Conclusions

All the four GPP roles were being reflected in the FIP annual congress programmes, both in the decade preceding their publication and in the decade after their publication. The more pronounced dominance toward the roles related to improving professional performance and positioning pharmacy are in line with the trend that the more technical topics in pharmacy are increasingly covered by specialised meetings and that the FIP annual congresses have moved toward more general, scholarly platforms for dialogue and conversation.

## Data Availability

The datasets analysed during the current study are available from the corresponding author on reasonable request and with permission of the International Pharmaceutical Federation.
